# Effects of hyperglycemia and intensive insulin therapy on neurons and glial cells during critical illness

**DOI:** 10.1186/cc9810

**Published:** 2011-03-11

**Authors:** R Sonneville, H Den Hertog, F Güiza, I Derese, JP Brouland, F Gray, F Chrétien, T Sharshar, D Annane, G Van den Berghe, I Vanhorebeek

**Affiliations:** 1 KU Leuven, Belgium; 2 Lariboisière University Hospital, Paris, France; 3 Pasteur Institute, Paris, France; 4 Raymond Poincare University Hospital, Garches, France

## Introduction

Treating hyperglycemia with intensive insulin therapy (IIT) may improve outcome of critically ill patients. However, this benefit may be counteracted by the increased risk of hypoglycemic episodes with this intervention, which may cause brain damage. We determined the effects of hyperglycemia and IIT on neurons and glial cells during critical illness.

## Methods

We performed a postmortem examination of the hippocampus and frontal cortex of 10 critically ill patients who were randomized to conventional insulin therapy (CIT, *n *= 5) or IIT (*n *= 5) in two previous studies [1,2]. Glucose levels differed between CIT (9.3 (8.5 to 11.2) mmol/l) and IIT (6.1 (5.3 to 6.2) mmol/l) patients (*P *< 0.01). Neuronal damage and density and function of glial cells were assessed by histochemistry and western blot. Data were compared with eight age-matched controls who died suddenly from extracranial injury. Mechanisms were explored in a validated burn injury model of prolonged critical illness. Critically ill rabbits were allocated to four groups, each a combination of normal or elevated blood glucose with normal or elevated insulin levels. Brain samples were collected after 7 days of illness. Healthy rabbits were included as controls.

## Results

In the hippocampus of CIT patients, neuronal damage (*P *= 0.002) and microglia activation (*P *= 0.003) were increased as compared with controls. Density (*P *= 0.02) and activation status (*P *= 0.03) of astrocytes were decreased. IIT did not affect neuronal damage, but reduced microglia activation (*P *= 0.03) and restored astrocyte function and density (*P *= 0.009) versus CIT. Findings in the frontal cortex were largely similar. The experimental model showed pronounced neuronal damage and microglia activation with hyperglycemia, which were restored to normal levels with normoglycemia. Astrocytes were activated only in rabbits with high insulin and normal glucose levels, without increased network formation, as assessed by connexin-43 levels. MnSOD protein expression levels suggested reduced oxidative stress by glycemic control under high insulin levels.

## Conclusions

Critical illness is characterized by increased neuronal damage and microglia activation in the hippocampus and frontal cortex under hyperglycemia. Our data suggest that maintaining normoglycemia with IIT reduces brain inflammation and may be neuroprotective, despite the risk of brief episodes of severe hypoglycemia.
